# Supplementary material for: Synthetic urine oversimplification results in misleading membrane fouling mechanisms in bipolar membrane electrodialysis
Source: Nat Commun. 2026 Apr 10;17:3395. doi: 10.1038/s41467-026-70034-w (PMC13069111; doi:10.1038/s41467-026-70034-w)
Supplement: Supplementary file 1 — Supporting Information [file 41467_2026_70034_MOESM1_ESM.pdf]

Supplementary Information for

Synthetic urine oversimplification results in misleading membrane fouling mechanisms in bipolar membrane electrodialysis

Hao-Ran Yang [1, 2], Shu-Jie Hu [1, 2], Meng-Yue Zhang [1, 2], Di Wu [1, 2], Lei Zheng [1, 2], Qianhong She [3, 4], Zhi-Hua Yuan [5], Lin-Jiang Zhong [1, 2], Xuan Zhao [6], Ying Chen [6], Hong Liu [1, 2], Lin-Ji Xu [7, *], Yuan Liu [1, *]

[1] State Key Laboratory of Lake and Watershed Science for Water Security, Chongqing Institute of Green and Intelligent Technology, Chinese Academy of Sciences, Chongqing 400714, China

[2] Chongqing School, University of Chinese Academy of Sciences, Chongqing 400714, China

[3] School of Civil and Environmental Engineering, Nanyang Technological University, 50 Nanyang Avenue, Singapore 639798, Singapore

[4] Singapore Membrane Technology Centre, Nanyang Environment and Water Research Institute, Nanyang Technological University, 1 Cleantech Loop, Clean Tech One, #06-08, Singapore 637141, Singapore

[5] CAS Key Laboratory of Urban Pollutant Conversion, Institute of Urban Environment, Chinese Academy of Sciences, Xiamen 361021, China

[6] School of Environmental Science and Engineering, Southwest Jiaotong University, Chengdu 610031, China

[7] College of Environment and Ecology, Chongqing University, Chongqing 400044, China

***Co-corresponding Authors**

*Yuan Liu: liuyuan@cigit.ac.cn

*Lin-Ji Xu: lin_ji_good@126.com

Table of Contents

Supplementary Methods 3

Supplementary Notes.. 10

Supplementary Figures 12

Supplementary Tables.. 24

Supplementary References.. 31

Supplementary Methods

Supplementary Method 1: Urea self-aggregation: A theoretical calculation study

Experimental observations revealed that urea aggregates form near-spherical structures with an average diameter of 1.4 μm. Furthermore, experimental characterization confirmed that the self-aggregation of urea is primarily driven by hydrogen bonding, with other forces playing a synergistic role. Here, we present a theoretical framework to elucidate the forces governing the stability of these aggregates, offering insights into the underlying mechanisms.

According to previous research[1,2], a single urea molecule has a diameter of approximately 0.4 nm and a radius of 0.2 nm. Based on these dimensions, the volume of a spherical urea molecule can be approximated as $V_{urea} = 3.35 \times 10^{-29}\ m^3$.

In this context, urea molecules undergo self-aggregation to form aggregates. Assuming the aggregates were perfect spheres with a diameter of 1.4 μm and a radius r = 0.7 μm, the surface area and volume of such an aggregate were calculated as $A_{sphere} = 6.157 \times 10^{-12}\ m^2$, and $V_{sphere} = 1.436 \times 10^{-18}\ m^3$, respectively. Using these values, the total number of urea molecules (N_{total}) within the aggregate can be calculated as shown in Equation 1.

$$N_{total} = \frac{V_{sphere}}{V_{urea}} = 4.285 \times 10^{10}\ molecules \qquad (1)$$

Through multimodal characterization techniques, it was observed that the aggregation of urea was predominantly driven by hydrogen bonding interactions (Figs. 2 and 3). To investigate the contribution of hydrogen bonds to urea aggregation, we calculated the total hydrogen-bond interaction energy ($E_{hb,total}$) within the aggregate, as shown in Equation 2.

The hydrogen bonding interactions of urea are determined by its molecular structure: each urea molecule has one carbonyl oxygen atom (C=O) as potential hydrogen bond acceptors and four hydrogens on the two amino groups ($-NH_2$) as donors, theoretically allowing for up to four hydrogen bonds per molecule. However, in actual aggregates, spatial constraints limit this number, often resulting in only two effective hydrogen bonds

per molecule (e.g., each carbonyl oxygen interacts with two hydrogens from adjacent molecules). In acidic conditions, hydrogen bonding strength can increase by 5-15% due to proton-mediated effects of bipolar membrane electrodialysis (BMED)[3,4]. In the context of BMED processes studied here, proton leakage provided an environment conducive to this enhancement[5]. This study assumed a 10% enhancement in hydrogen bond strength, consistent with experimental observations and facilitating more accurate calculations.

$$E_{hb,total} = N_{total} \times 2 \times E_{hb} = 1.57 \times 10^{-9}\,J \tag{2}$$

where $E_{hb,total}$ represents the total hydrogen-bond interaction energy within the aggregate, E_{hb} is the enhanced hydrogen-bonding energy per urea molecule ($1.83 \times 10^{-20} J$) after considering a 10% proton- mediated enhancement, and N_{total} denotes the total number of urea molecules in the aggregate.

Notably, the stability of urea aggregates must overcome both thermal energy ($E_{thermal}$) and shear forces (E_{shear}) generated during BMED processing. Thermal energy plays a critical role in influencing molecular motion and interactions[6,7], thereby affecting the formation and stability of aggregates. To quantify this effect, we employed the total thermal energy formula (Equation 3) to calculate the average thermal energy across all molecules in the system and examined how temperature and molecular population influence the overall energy distribution.

$$E_{thermal} = N_{total} \times k_B \times T = 1.77 \times 10^{-10}\,J \tag{3}$$

where k_B represents the Boltzmann constant (1.38×10^{-23} J K^{-1}) and T is the temperature of the system in kelvin (with a value of 298 K in this study).

Shear force energy (E_{shear}) refers to the energy required for aggregates to overcome shear stress (τ) during fluid flow[8]. Specifically, E_{shear} represents the energy dissipation resulting from interactions between the fluid and aggregates, as described by Equation 4.

$$\tau = \frac{\mu \times u}{h} \tag{4}$$

where (u) represents the fluid velocity (1.21×10^{-3} m s^{-1}), μ denotes the dynamic viscosity (1.07×10^{-3}, Pa·s), and h is the characteristic fluid length scale (1×10^{-6} , m)—here taken as the typical thickness of fluid adjacent to a membrane surface. Notably, the

aggregates are stable and have not shifted, thus d = 0.

Shear force energy (E_{shear}) is calculated using Equation 5:

$$E_{shear} = \tau \times A_{sphere} \times d = 0\,J \quad (5)$$

Since urea formed stable aggregates on ion exchange membranes with a diameter of approximately 1.4 μm, the total hydrogen-bonding energy ($E_{hb,total}$) must meet certain conditions to ensure aggregate stability. The total bonding energy required for these aggregates was determined by balancing the contributions from hydrogen bonding and other forces acting on the system (Equation 6).

$$E_{hb,total} \geq \alpha \times (E_{shear} + E_{thermal}) \quad (6)$$

The stability coefficient α is introduced to account for potential destabilizing effects caused by random perturbations in the environment, such as flow fluctuations and instantaneous energy changes due to molecular motion. In addition to thermal energy ($E_{thermal}$) and shear forces (E_{shear}), aggregates may experience transient disruptive energies exceeding average values. Thus, introducing α provides a margin of safety for bonding energy, ensuring aggregate stability under dynamic conditions. Furthermore, in the theoretical calculations of this study, since E_{shear} and $E_{thermal}$ are based on simplified estimates, α helps compensate for associated approximations. Based on principles of colloid stability[9], a value of $\alpha = 10$ is employed to ensure that bonding energy significantly exceeds perturbation energies in the environment. This choice aligns with the Boltzmann distribution criterion[10-12] $P_{destruction} \propto exp(-\alpha\Delta E/K_BT)$, where $\alpha = 10$ reduces the destruction probability below the thermodynamic stability threshold (10^{-10}). The energy scale of this system is similar to previously reported research[2], further validating the rationality of α. However, the calculation revealed that $1.57 \times 10^{-9}\,J \geq 10 \times (1.77 \times 10^{-10} + 0)$, which is inconsistent with fundamental principles.

It is important to note that while hydrogen bonding exhibits strong directional and attractive properties, solely analyzing the energy comparison reveals that the hydrogen-bonding energy is insufficient compared to the required energies for disruption (i.e., shear forces and thermal energy). This indicates that hydrogen bonding alone cannot sustain

aggregate stability. However, the formation of hydrogen bonding networks compresses molecular distances into the range where van der Waals interactions become significant (0.3–0.6 nm), thereby amplifying their cumulative effects[13-16]. Molecular dynamics simulations have confirmed this conclusion (Fig. 2d-e). Additionally, in acidic conditions, hydrophobic interactions further contribute to the stability of aggregates[17,18]. Together, these findings suggest that van der Waals forces and hydrophobic interactions are critical supplementary mechanisms for maintaining the stability of urea aggregates.

Van der Waals forces, as short-range molecular interactions, are typically confined to a critical distance range of 0.3–0.6 nm[16]. Under these high-density conditions, each urea molecule may interact with 6–12 neighboring molecules, consistent with the close-packing model for spherical particles. In this study, we adopted the modified face-centered cubic packing model proposed in previous research[19], assuming that each urea molecule forms van der Waals interactions with six nearest neighbors. This coordination number was selected based on structural loosening caused by molecular thermal motion in solution[20]. Consistent findings have been reported in previous studies[2], further validating this approach. The energy of a single van der Waals interaction is calculated using the Lennard-Jones potential function (Equation 7):

$$E_{vdW} = 4 \in [(\frac{\sigma}{r})^{12} - (\frac{\sigma}{r})^{6}] \quad (7)$$

where $\in$ represents the depth of the potential well ($3.3 \times 10^{-21}\, J$)[2], σ is the distance at which the potential equals zero (0.4 nm), and r is the intermolecular separation (0.5 nm), derived from the half-peak width of the radial distribution function obtained via molecular dynamics simulations

The total van der Waals interaction energy ($E_{vdW,total}$) can be calculated using Equation 8.

$$E_{vdW,total} = \frac{1}{2} N_{total} \times 6 \times E_{vdW} = -3.28 \times 10^{-10} \quad (8)$$

The calculation incorporates a symmetry correction (division by two) and solution-phase molecular separation parameters, providing a more accurate representation of real systems compared to traditional close-packing models (e.g., $N_n = 12$). Furthermore, the

negative value of the van der Waals force calculation confirms its attractive nature, as the Lennard-Jones potential dictates that intermolecular interactions are attractive when the separation distance exceeds the zero-potential threshold σ.

Previous study[16] has reported that the surface free energy of molecules with hydrophobic characteristics in aqueous solutions typically ranges from 0.03 J m^{-2} to 0.07 J m^{-2}. Although urea is inherently a polar molecule, its amino and carbonyl groups can form internal hydrogen bonding networks. In acidic conditions (e.g., pH 3-4), these molecular interactions lead to the exclusion of water molecules, resulting in a degree of hydrophobicity. Consequently, the total hydrophobic interaction energy ($E_{hydrophobic,total}$) associated with urea aggregation can be calculated using Equation 9.

$$E_{hydrophobic,total} = \gamma \times A_{sphere} = 3.08 \times 10^{-13}\, J \tag{9}$$

where $\gamma = 0.05$ J m^{-2} represents the hydrophobic surface free energy associated with urea aggregation under these conditions.

Consequently, the total bonding energy was the sum of contributions from hydrogen bonding, van der Waals interactions, and hydrophobic effects, as shown in Equation 10.

$$E_{total} = E_{hb,total} + |E_{vdW,total}| + E_{hydrophobic,total} = 1.90 \times 10^{-9}\, J \tag{10}$$

Based on calculations, the total bonding energy exceeds the energies associated with other interactions, indicating that the aggregates could remain stable under these conditions (as shown in Equation 11).

$$E_{total} \geq \alpha \times (E_{shear} + E_{thermal}) \tag{11}$$

In conclusion, the stability of urea aggregates arose from synergistic contributions of hydrogen bonding, van der Waals forces, and hydrophobic interactions. The latter two mechanisms provided critical reinforcement under acidic BMED conditions, compensating for the limitations of hydrogen bonds alone. This multi-mechanism framework aligns with prior studies on urea aggregation in aqueous systems and underscores the importance of interfacial chemistry in ion-exchange membrane processes.

Supplementary Method 2: Prediction of membrane cleaning frequency in BMED-HFM system.

Experimental integrity in wastewater treatment studies hinges on the faithful representation of real-world effluent complexity. This study demonstrates that oversimplifying wastewater formulation—exemplified by Group A (urea-only)—fundamentally distorts the interpretation of membrane fouling mechanisms. While the performance decay of Group A was adequately described by an asymptotic model ($y = a - b \times c^x$, R^2 = 0.99159), this exponential form presupposes a unidirectional decline toward equilibrium, neglecting dynamic interactions inherent to multicomponent systems (Supplementary Fig. 11 and Supplementary Table 4). For instance, the model reduces fouling dynamics to a rate constant (c), ignoring transient phenomena such as competitive adsorption-desorption or colloidal destabilization. In contrast, Groups B and C (containing antagonistic constituents) required a fifth-order polynomial (Poly5, $R^2 \approx 0.9968$ and 0.9967), where higher-order terms (e.g., x^3, x^5) captured multistage nonlinear decay. This divergence underscores that simplified compositions obscure synergistic or antagonistic interactions, leading to flawed predictions of long-term fouling behavior.

The practical implications of formulation simplification extend to maintenance strategy misjudgments. At a 50% performance threshold, Group A demanded cleaning at 6.33 batches, whereas Groups B and C delayed intervention to 7.49 and 7.50 batches, respectively. This delay suggests antagonistic components (e.g., organic matter in Groups B and C) mitigate fouling via mechanisms such as site competition or dispersion effects, consistent with characterization results (Fig. 2 and 3). The model of Poly5 higher-order coefficients (e.g., f = 0.14392 in Group C) further revealed oscillatory fouling rates, indicative of dynamic equilibria between deposition and mitigation. Reliance on Group A's simplified model risks premature cleaning cycles, inflating operational costs and artificially truncating membrane lifespan. Such discrepancies highlight a critical pitfall: idealized experimental designs yield strategies misaligned with the heterogeneous realities of industrial wastewater.

Mechanistically, the advantage of complex compositions lies in their capacity to establish self-limiting fouling regimes. For Groups B and C, the significance of fifth-order terms implies emergent "self-buffering" behavior, where cumulative fouling triggers intermolecular repulsion or steric hindrance—phenomena absent in Group A's monotonic decay. Parameter analysis (e.g., $c = -94.68$ and $d = 24.98$ in Group C) further suggests that antagonistic constituents promote reversible adsorption, forming loose cake layers that reduce irreversible fouling. Oversimplified formulations, however, mistakenly attribute membrane performance to singular pollutant characteristics, ignoring the interplay of multicomponent systems. This oversight risks mischaracterizing membrane antifouling potential and misguiding material selection for real-world applications.

In conclusion, oversimplification of wastewater formulation not only diminishes component fidelity but also confines conclusions to artificial scenarios. This study establishes that multicomponent interactions—quantifiable through higher-order models—critically regulate fouling dynamics, while asymptotic approximations overstate degradation rates and undervalue membrane resilience. Future research should prioritize enhancing the representativeness of synthetic wastewater compositions and develop adaptive modeling frameworks to bridge the gap between lab-scale synthetic wastewater studies and real-world industrial wastewater applications.

Supplementary Notes

Supplementary Note 1: Analysis of hydroxide scaling formation on membrane surfaces

The spatial distribution of inorganic scales on the membrane surfaces, as revealed by SEM-EDS elemental mapping (Supplementary Figs. 9 and 10), provides direct evidence for the formation of distinct hydroxide deposits. The mapping shows a clear spatial correlation between Ca and O on the anion-exchange side of the bipolar membrane (BPM), and a concurrent correlation between Mg and O on the base chamber-side surface of the cation exchange membrane (CEM). Critically, the absence of a nitrogen signal in these deposits definitively excludes nitrogen-containing scales such as struvite ($MgNH_4PO_4·6H_2O$, MAP). Furthermore, the system design inherently excludes phosphate from the base chamber, as phosphate anions electromigrate through the anion exchange membrane (AEM) toward the acid chamber. This elemental co-localization, in the absence of other scaling precursor ions, identifies the primary deposits as $Ca(OH)_2$ and $Mg(OH)_2$, respectively.

The divergent scaling locations are a direct result of the differential mobility of Ca^{2+} and Mg^{2+} under the applied electric field. Mg^{2+}, with its high charge density and strong hydration energy (-1922 kJ mol^{-1}), interacts more strongly with the CEM surface and possesses lower mobility. This results in its preferential accumulation and rapid precipitation as $Mg(OH)_2$ upon encountering the high-pH environment at the CEM surface, effectively hindering its further transport. In contrast, the more weakly hydrated Ca^{2+} (hydration energy: -1577 kJ mol^{-1}) exhibits greater mobility, facilitating its transit across the CEM[21,22]. Once in the base chamber, Ca^{2+} is further driven towards the BPM by the electric field, where it encounters the most concentrated OH^- flux and precipitates as $Ca(OH)_2$ on the BPM's anion-exchange layer.

In conclusion, the scaling pattern is not stochastic but is a deterministic outcome of the system's electrochemistry. The combined evidence from elemental mapping and thermodynamic analysis confirms that the observed deposits are $Ca(OH)_2$ and $Mg(OH)_2$,

whose formation sites are kinetically controlled by the differential migration of the hardness ions toward a pervasive high-pH sink.

Supplementary Figures

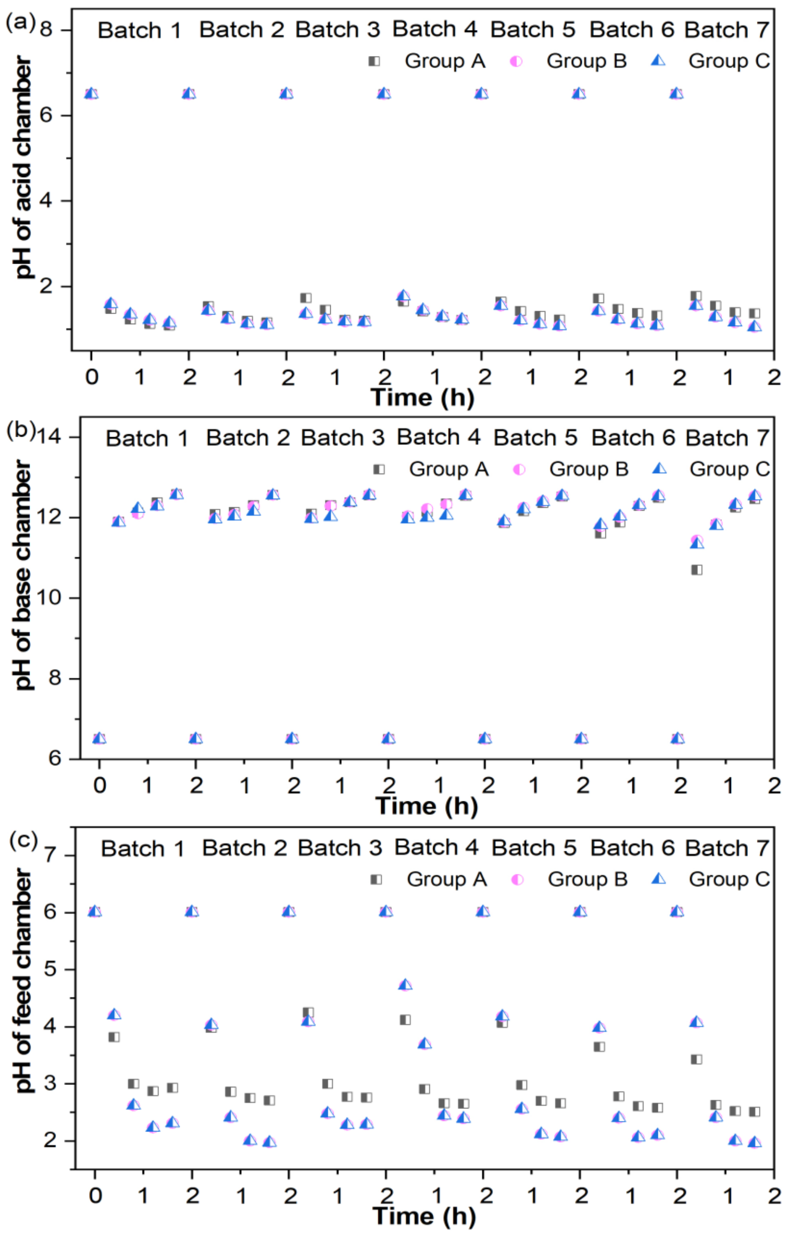

Supplementary Fig. 1 | pH in BMED chambers during urine treatment over 7 batches: (a) acid, (b) base, and (c) feed chambers.

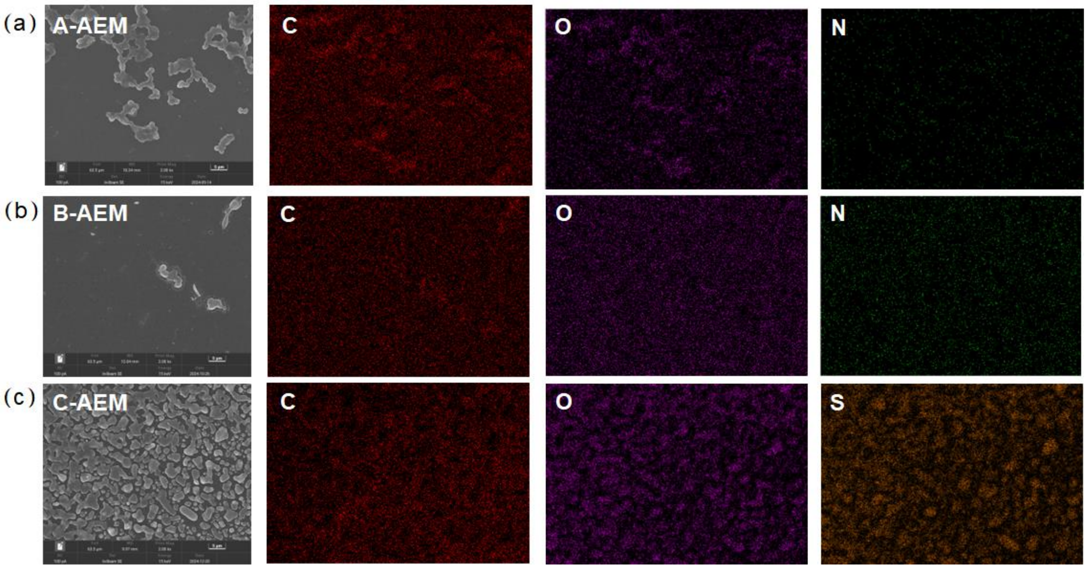

Supplementary Fig. 2 | SEM morphological and elemental mapping analysis of AEM treated with various formulated urines by BMED system: **a** A-AEM (treated with urine of Group A), **b** B-AEM (treated with urine of Group B), **c** C-AEM (treated with urine of Group C).

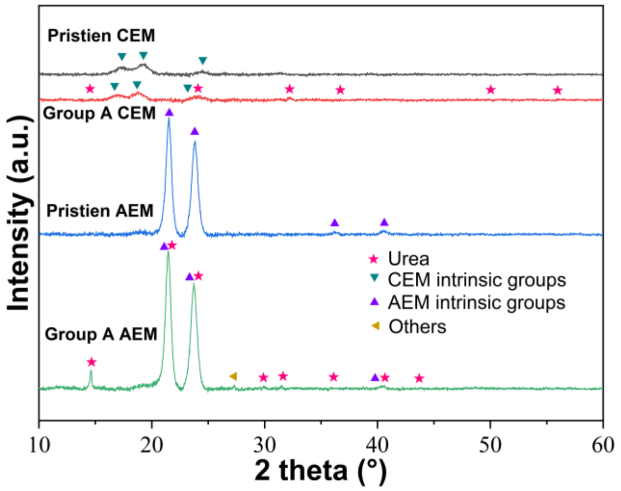

Supplementary Fig. 3 | X-ray diffraction patterns of cation-exchange membranes (CEM) and anion-exchange membranes (AEM) before and after fouling during the treatment of Group A urine using bipolar membrane electrodialysis (BMED). The analysis was performed using a Rigaku Ultima IV diffractometer (Japan). Peaks corresponding to urea molecules were identified based on previous studies[23-25], indicating that the fouling deposits on the membranes predominantly consist of urea. The patterns are categorized into Pristine CEM, Group A CEM, Pristine AEM, and Group A AEM, with intrinsic groups and other components also noted. The 2θ range from 10° to 60° is shown, highlighting the characteristic peaks of urea.

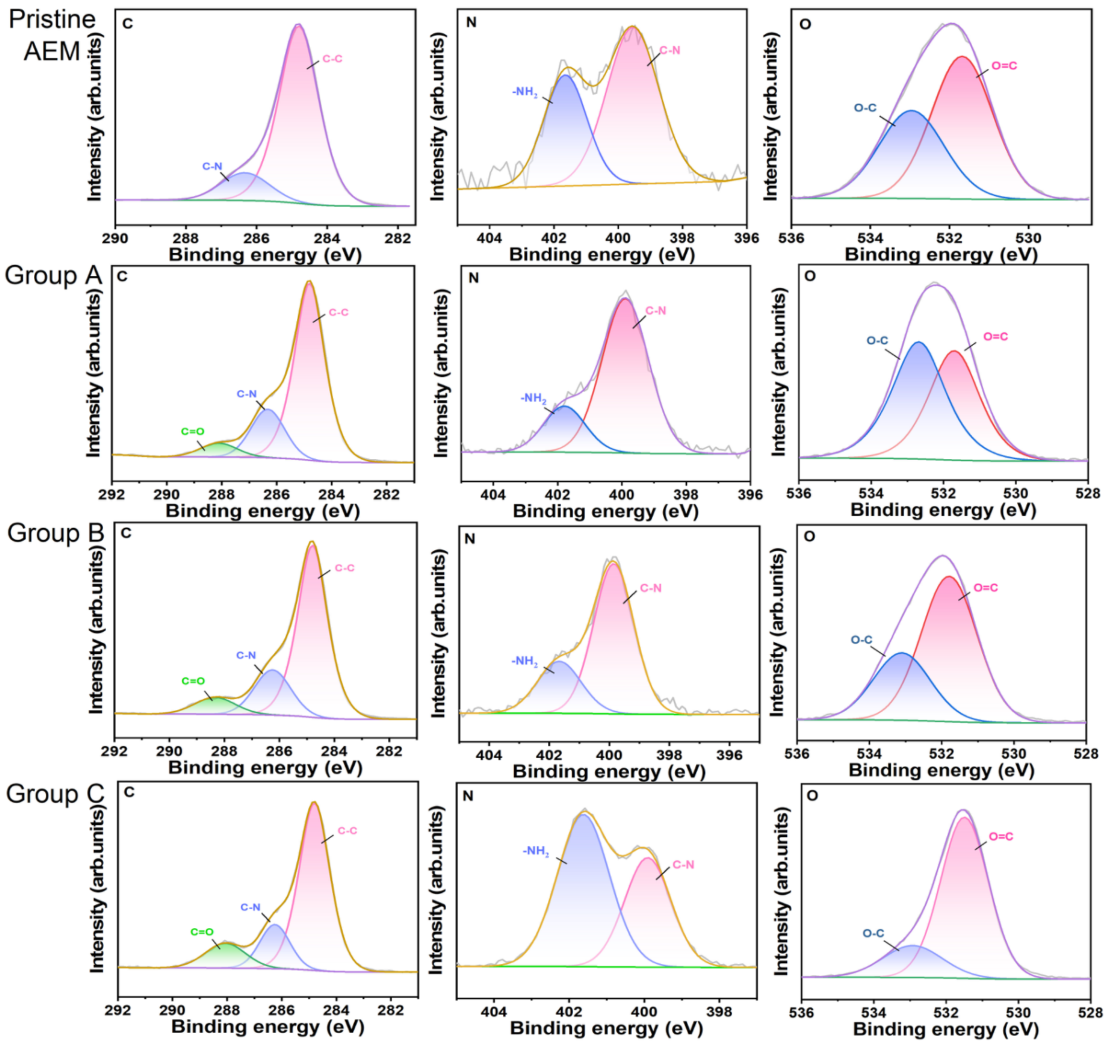

Supplementary Fig. 4 | XPS characterization of C-N-O functional groups on AEM surfaces: Fine spectra of pristine, Group A, Group B, and Group C after BMED of different urine components.

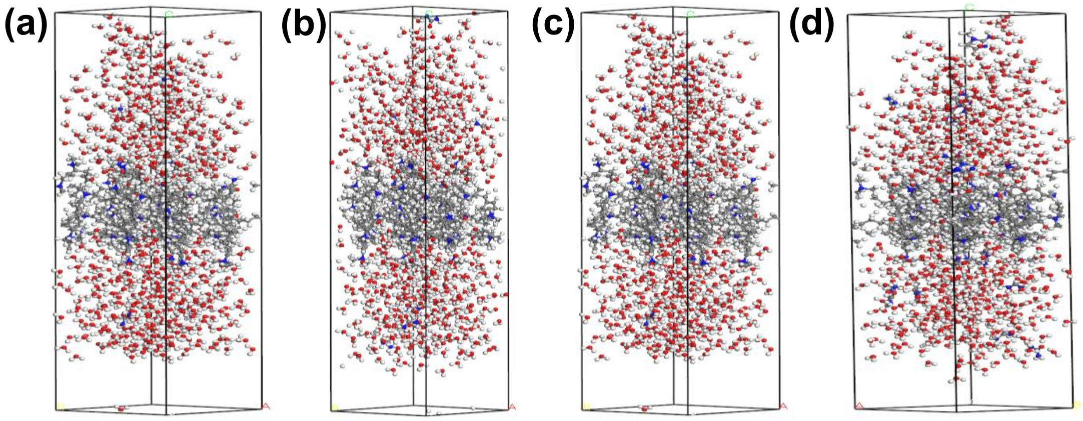

Supplementary Fig. 5 | Molecular dynamics simulation snapshots of AEM-urea and AEM-urea-mixed systems: (a) Optimized AEM-urea (optimized initial structural model); (b) 600 ps AEM-urea (configuration after 600 ps simulation); (c) Optimized AEM-urea-mixed (optimized initial structural model); (d) 600 ps AEM-urea-mixed (configuration after 600 ps simulation). The snapshots are coupled with molecular structure depictions of H_2O, urea, uric acid, creatinine, and AEM. The specific molecular structure is shown below:

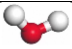	H_2O
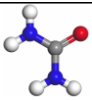	Urea
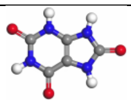	Uric acid
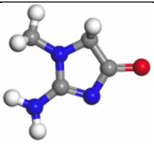	Creatinine
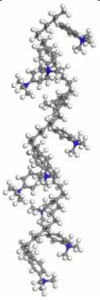	AEM functional group

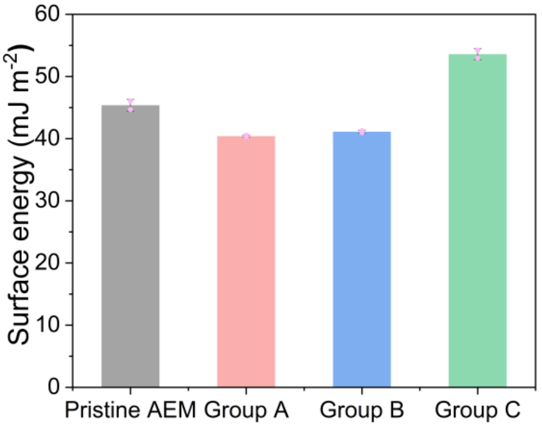

Supplementary Fig. 6 | Surface energy measurements of AEM Fouling in a BMED system during nutrient recovery from urine across multiple cycles. Error bars represent the standard deviation (s.d.) calculated from n = 2 measurements taken at different locations on each membrane surface to account for spatial heterogeneity. AEM, anion-exchange membrane; BMED, bipolar membrane electrodialysis.

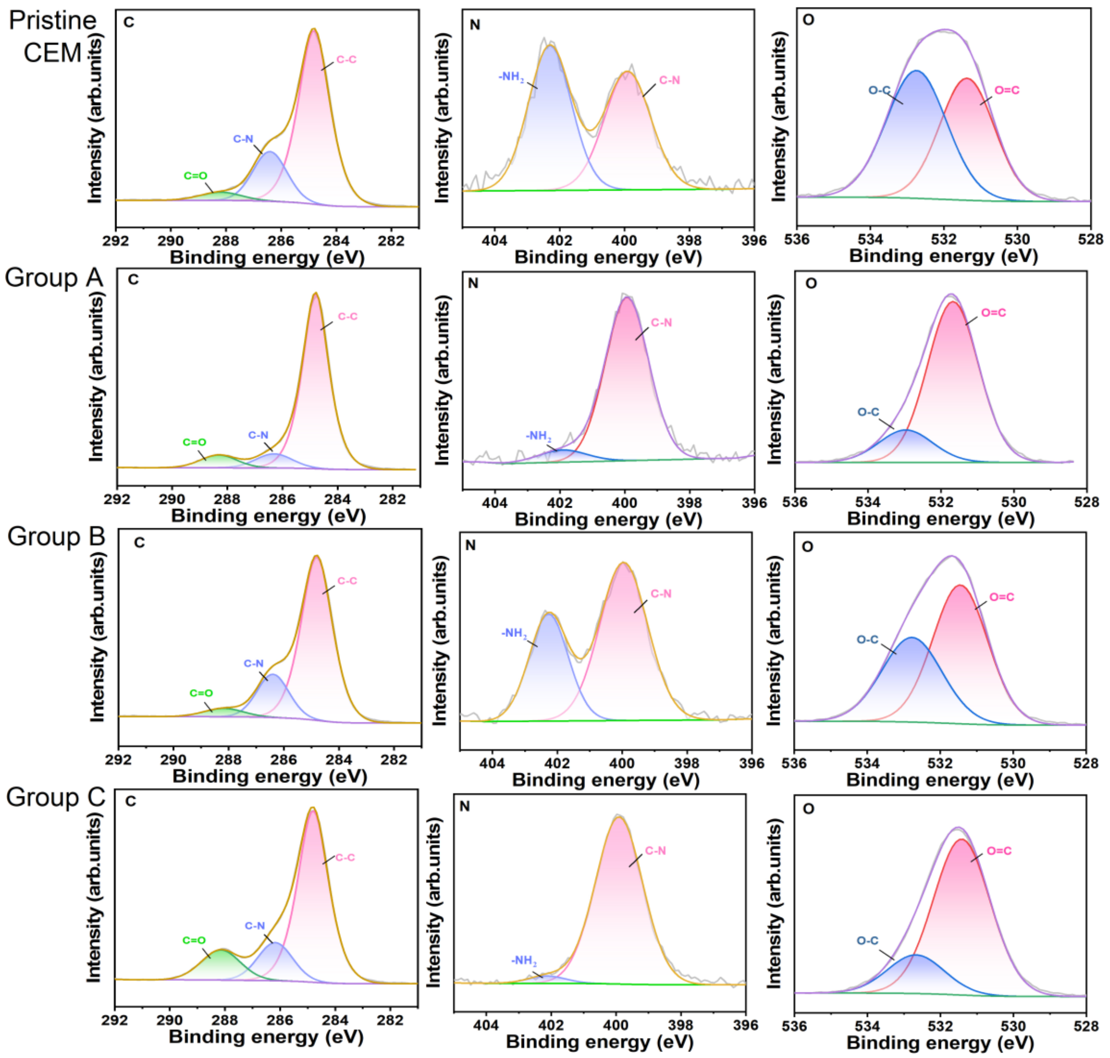

282

283 **Supplementary Fig. 7 |** XPS characterization of C-N-O functional groups on CEM
284 surfaces: Fine spectra of pristine, Group A, Group B, and Group C after BMED of different
285 urine components.

286

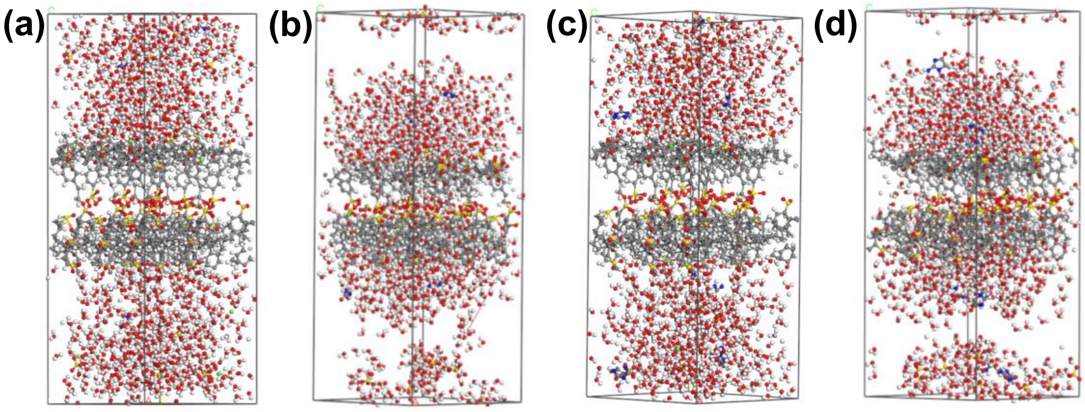

Supplementary Fig. 8 | Molecular dynamics simulation snapshots of CEM-urea and CEM-urea-mixed systems: (a) Optimized initial structural model of CEM-urea; (b) 600 ps simulation configuration of CEM-urea; (c) Optimized initial structural model of CEM-urea-mixed; (d) 600 ps simulation configuration of CEM -urea-mixed. The snapshots incorporate molecular structure depictions of H_2O, Ca^{2+}, Mg^{2+}, urea, uric acid, creatinine, and CEM. The specific molecular structure is shown below:

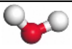	H_2O
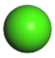	Ca^{2+}
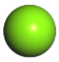	Mg^{2+}
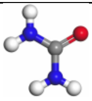	Urea
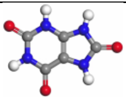	Uric acid
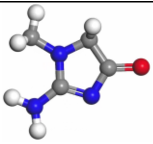	Creatinine
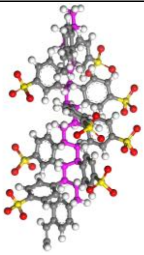	CEM functional group

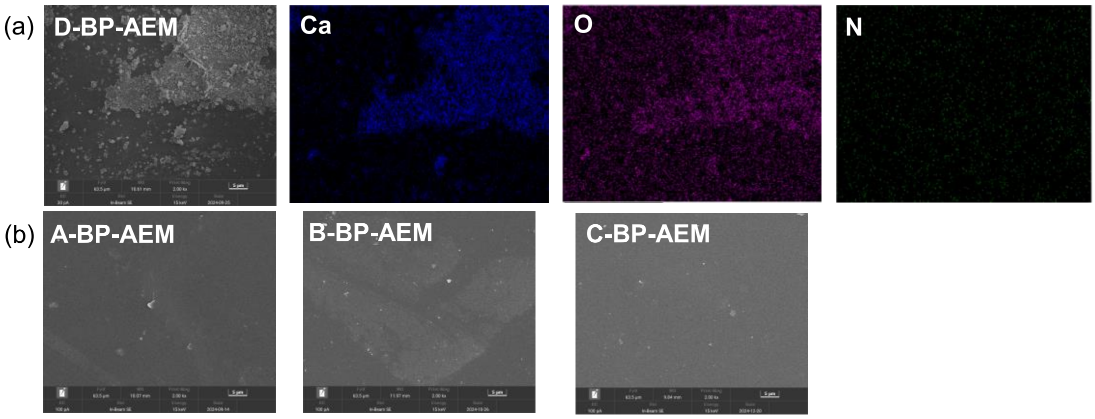

Supplementary Fig. 9 | a Surface mapping analysis after treatment with Group D. BP-AEM: Cathode side of the bipolar membrane, i.e., the OH^- production side and **b** Contamination characteristic of BP-AEM surfaces after seven batches of BMED treatment with different urine components (Groups A to C).

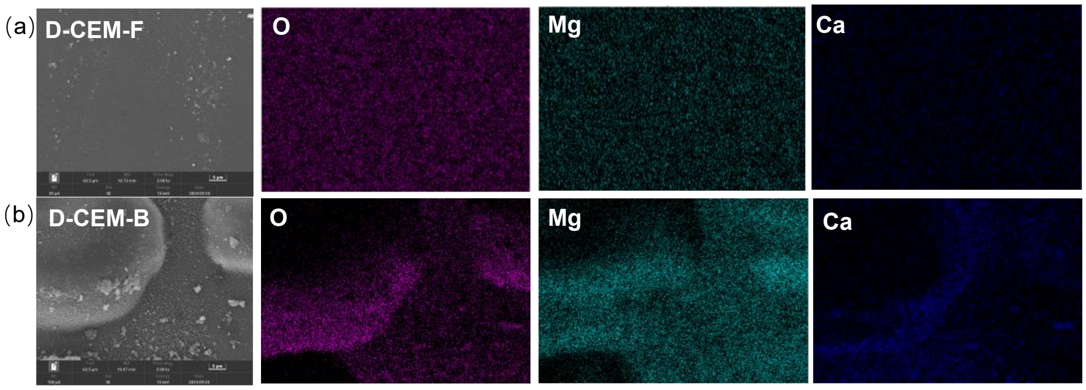

Supplementary Fig. 10 | SEM imaging and elemental mapping analysis of CEM in the Group D after BMED treatment of seven urine batches. The images show the morphology of **a** urine side and **b** base side.

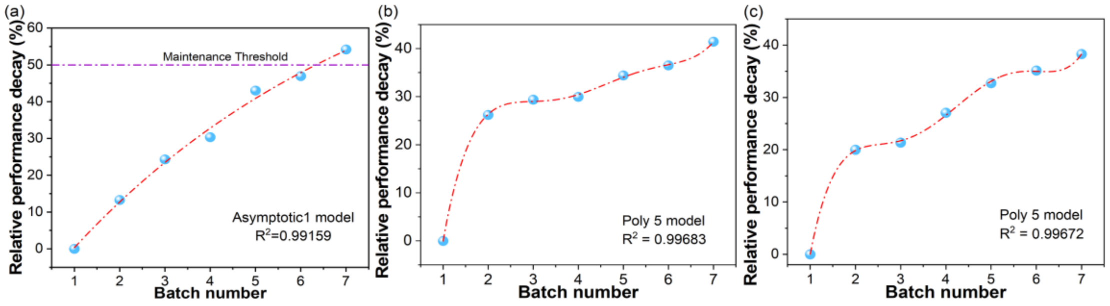

Supplementary Fig. 11 | Performance degradation prediction model for BMED treatment applied to urine with varying compositions. **a** Analysis of Group A, **b** Analysis of Group B, and **c** Analysis of Group C. The experimental data are fitted using an Asymptotic1 model for Group A (R^2 = 0.99159) and Poly 5 models for Group B (R^2 = 0.99683) and Group C (R^2 = 0.99672). The horizontal dashed line in a indicates the 50% maintenance threshold. Model parameters are detailed in Supplementary Table 4.

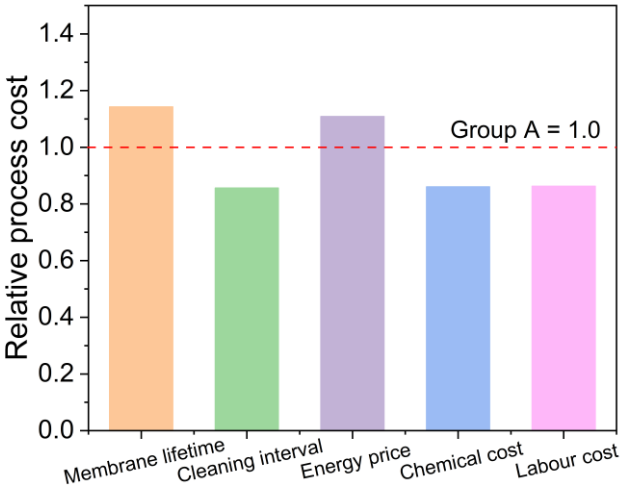

Supplementary Fig. 12 | Relative process cost (Group C vs. Group A = 1.0). This chart illustrates the relative cost of each major cost component for Group C compared to the Group A baseline (red line = 1.0). The values are calculated based on the cost data in Supplementary Table 3 and the parameter ranges defined in Supplementary Table 5. While Group C incurs a higher total energy cost (ratio > 1.0), this is significantly offset by its superior performance. The longer membrane lifespan and extended cleaning interval result in lower costs for membrane replacement (fixed costs), chemicals, and labour (all other cost ratio < 1.0)

Supplementary Tables

Supplementary Table 1 | Typical concentration ranges of major components in human urine.

Composition	Normal range in humans
Urea (CH_4N_2O)	10–35 g d^{-1}
Uric acid ($C_5H_4N_4O_3$)	<750 mg d^{-1}
Creatinine ($C_4H_7N_3O$)	Males: 955-2936 mg d^{-1}<br>Females: 601-1689 mg d^{-1}
Citrate ($C_6H_5O_7^{3-}$)	221-1191 mg d^{-1}
Sodium (Na^+)	41-227 mmol d^{-1}
Potassium (K^+)	17-77 mmol d^{-1}
Ammonium (NH_4^+)	15-56 mmol d^{-1}
Calcium (Ca^{2+})	Males: < 250 mg d^{-1}<br>Females: < 200 mg d^{-1}
Magnesium (Mg^{2+})	51-269 mg d^{-1}
Chloride (Cl^-)	40-224 mmol d^{-1}
Oxalate ($C_2O_4^{2-}$)	0.11-0.46 mmol d^{-1}
Sulphate (SO_4^{2-})	7-47 mmol d^{-1}
Phosphate (PO_4^{3-})	20-50 mmol d^{-1}

Note: Data are compiled from reference[26,27].

Supplementary Table 2 | Components of substances in urine for each group.

Components (g L^{-1})	Group A	Group B	Group C	Group D
Na_2SO_4	1.7	1.7	1.7	1.7
$Na_3C_6H_5O_7·2H_2O$	0.72	0.72	0.72	0.72
KCl	2.308	2.308	2.308	2.308
NaCl	1.756	1.756	1.756	1.756
$CaCl_2$	0.185	0.185	0.185	0.185
NH_4Cl	1.266	1.266	1.266	1.266
$K_2C_2O_4·H_2O$	0.035	0.035	0.035	0.035
$MgSO_4·7H_2O$	1.082	1.082	1.082	1.082
$NaH_2PO_4·2H_2O$	2.912	2.912	2.912	2.912
$Na_2HPO_4·2H_2O$	0.831	0.831	0.831	0.831
Urea	15	15	15	-
Uric acid	-	0.05	0.05	-
Creatinine	-	0.881	0.881	-
BSA	-	-	0.08	-

Note: The concentrations of components in all formulations were designed to reflect typical levels found in real human urine. The specific organic metabolites (urea, uric acid, creatinine) and model protein (BSA) were included at concentrations within their physiological ranges documented in the literature (Supplementary Table 1). Inorganic salt concentrations were calculated to match the ionic strength and elemental composition (e.g., Na^+, K^+, NH_4^+, Cl^-, SO_4^{2-}) of real urine.

Supplementary Table 3 | Engineering-economic assessment of resource recovery from urine using a BMED system across different groups.

Items	Parameters	Specifications	Group		
			A	B	C
I. Operation conditions	Applied voltage, V	−		15	
	C: Cleaning cycle, batches	−	6	7	7
	A: Effective membrane area, cm^2	−		55	
	F: Flow rate, L min^{-1}	−		0.4	
	N: Repeating units	−		3	
	T: Batch experiment time, h	−		2	
	$\boldsymbol{T_d}$: Cleaning cycle duration, h	$T_d = C \times T$	12	14	14
	$\boldsymbol{T_f}$: Cleaning frequency, year	Annual operation days = 330	634	547	547
II. Product yield	$\boldsymbol{Y_b}$: Base product yield, kg m^{-3}	−	3.84	4.18	4.31
	$\boldsymbol{Y_a}$: Acid product yield, kg m^{-3}	−	5.11	5.56	5.74
	$\boldsymbol{Y'_b}$: Base product yield, kg $year^{-1}$	−	2.19	2.4	2.47
	$\boldsymbol{Y'_a}$: Acid product yield, kg $year^{-1}$	−	2.91	3.19	3.29
	$\boldsymbol{Y_{rp}}$: Total product yield, kg m^{-3}	$Y_{rp} = Y_a + Y_b$	8.95	9.74	10.05
	$\boldsymbol{Y'_{rp}}$: Total product yield, kg $year^{-1}$	$Y'_{rp} = Y'_a + Y'_b$	5.1	5.59	5.76
III. Revenue from the product	$\boldsymbol{C_b}$: Income of base product, \$ $year^{-1}$	Market price [a]: 33 \$ t^{-1} 30% HCl, 550 \$ t^{-1} NaOH	1.2	1.32	1.36
	$\boldsymbol{C_a}$: Income of acid product, \$ $year^{-1}$		0.32	0.35	0.36
	$\boldsymbol{C_r}$: Income of product, \$ $year^{-1}$	$C_r = C_b + C_a$	1.52	1.67	1.72

	**E_c**: Average energy consumption, kW·h kg^{-1} base	–	25.97	26.54	25.66
	**E_t**: Total energy consumption, kW·h m^{-3}	$E_t = E_c \times Y_b$	99.72	110.937	110.595
	**P**: Process capacity, m^3 $year^{-1}$ [b]	Annual operation days = 330	0.57	0.57	0.57
IV. Energy cost	**C_e**: Electricity charge, \$ kW·h^{-1}	0.86 RMB		0.12	
	**C_{ep}**: Energy cost for production, \$ $year^{-1}$	$C_{ep} = C_e \times E_t \times P$	6.82	7.59	7.56
	**C_{ee}**: Energy cost for peripheral equipment, \$ $year^{-1}$	$C_{ee} = 0.05 \times C_{ep}$	0.34	0.38	0.38
	**C_{te}**: Total energy cost, \$ $year^{-1}$	$C_{te} = C_{ep} + C_{ee}$	7.16	7.97	7.94
	**T_t**: Cleaning time, h $year^{-1}$	$T_t = T_f \times 0.5$	317	273.5	273.5
	**C_c**: Chemical reagent price, \$ $year^{-1}$	Acidic cleaner: 33 \$ t^{-1} 30% HCl	7.63×10^{-5}	6.58×10^{-5}	6.58×10^{-5}
		Alkaline cleaner: 350\$ t^{-1} NaOH	2.66×10^{-3}	2.29×10^{-3}	2.29×10^{-3}
V. Clean cost	**C_{bd}**: Base loss from downtime, \$ $year^{-1}$	–	3.20×10^{-2}	3.00×10^{-2}	3.09×10^{-2}
	**C_{ad}**: Acid loss from downtime, \$ $year^{-1}$	–	1.34×10^{-2}	1.25×10^{-2}	1.3×10^{-2}
	**C_d**: Downtime cost [c], \$ $year^{-1}$	$C_d = C_{bd} + C_{ad}$	4.53×10^{-2}	4.26×10^{-2}	4.39×10^{-2}
	**C_l**: Labor cost, \$ $year^{-1}$	Labour charges: 2.74 \$ h^{-1}	868.58	749.39	749.39
	**C_{tc}**: Total clean cost, \$ $year^{-1}$	$C_{tc} = C_c + C_d + C_l$	868.63	749.44	749.44
VI. Fixed cost	**Y_m**: Membrane lifespan and amortization of the peripheral equipment [d], years	–	2.31×10^{-2}	2.64×10^{-2}	2.64×10^{-2}
	Bipolar membrane price, \$ m^{-2}	–		690.10	

	Ion exchange membrane price, \$ m^{-2}	–		132.67 (AEM), 207.3 (CEM)	
	$\boldsymbol{C_{fm}}$: Total Membrane cost, \$	–		20.79	
	$\boldsymbol{C_{tm}}$: Total Membrane cost, \$ $year^{-1}$	$C_m = C_{fm} / Y_m$	900	787.5	787.5
	$\boldsymbol{C_{fs}}$: Membrane stack cost, \$ $year^{-1}$	$C_{fs} = 0.6 \times C_{tm}$	540	472.5	472.5
	$\boldsymbol{C_{fe}}$: Peripheral equipment cost [e], \$ $year^{-1}$	$C_{fe} = 0.6 \times C_{fs}$	324	283.5	283.5
	$\boldsymbol{C_{fi}}$: Total fixed cost, \$ $year^{-1}$	$C_{fi} = C_{fs} + C_{fe} + C_m$	1764	1543.5	1543.5
VII. Actual process cost	$\boldsymbol{C_{ra}}$: Actual process cost, \$ $year^{-1}$	$C_{ra} = C_{te} + C_{tc} + C_{fi}$	2639.79	2300.91	2300.88
	$\boldsymbol{C_{ta}}$: Total process cost, \$ $year^{-1}$	$C_{ta} = C_{te} + C_{tc} + C_{fi} - C_r$	2638.27	2299.24	2299.16

Note: [a] Data were collected from the Alibaba.com.

[b] Lab-scale experiments are conducted with a per-batch processing capacity of 0.15 liters.

[c] The downtime maintenance period is set at half an hour.

[d] Based on the normal service life of the membrane (4 years) and a maintenance frequency of every three months, the lifespan of the lab-equipment membrane module and its depreciation cycle were calculated.

[e] The design life of the membrane stack and auxiliary equipment is 10 years.

Supplementary Table 4 | Model parameters for the performance degradation prediction of BMED treatment applied to urine samples with varying compositions.

Group	A	B	C
Model	Asymptotic1	Poly 5	Poly 5
Fitted equation	$y = a - b \times c^x$	$y = a + b \times x + c \times x^2 + d \times x^3 + e \times x^4 + f \times x^5$	$y = a + b \times x + c \times x^2 + d \times x^3 + e \times x^4 + f \times x^5$
a	93.39769 ± 19.41735	-104.43143 ± 11.8329	-98.14 ± 11.58617
b	107.38623 ± 16.84792	172.01438 ± 22.58013	170.80135 ± 22.1093
c	0.86668 ± 0.03856	-86.03292 ± 14.79683	-94.67542 ± 14.4883
d	-	20.7008 ± 4.32775	24.97943 ± 4.23751
e	-	-2.38133 ± 0.57949	-3.08489 ± 0.56741
f	-	0.10558 ± 0.02892	0.14392 ± 0.02832
R^2	0.99159	0.99683	0.99672
x (y=50, Maintenance threshold)	6.33	7.49	7.5
Cleaning period (batch)	6	7	7

Supplementary Table 5 | Key parameters and assumed ranges for economic sensitivity analysis of urine treatment via BMED.

Parameter	Baseline value	Variation range	Rationale for variation
Membrane lifetime	4 years	±50% (2–6 years)	The typical service life of industrial bipolar membranes is 3-5 years, with 6 years being the upper limit under ideal maintenance conditions.
Cleaning interval	Group A: 6 batches<br>Groups B/C: 7 batches	Group A: ±16.7%<br>Groups B/C: ±14.3%	In this study, the cleaning frequency is assumed to vary by ±1 batch from the baseline, based on experimental observations.
Energy price	0.12 $ kW·h^{-1}	±20%	Energy price fluctuations are often set at ±20% in international industrial contexts, reflecting regional differences and energy market movements.
Chemical cost	Acidic cleaner: 33 $ t^{-1}<br>Alkaline cleaner: 350 $ t^{-1}	±20%	The prices of acid and alkali reagents are influenced by raw material and transportation costs, and industry analysis often adopts a ±20% fluctuation.
Labour cost	2.74 $ h^{-1}	±15%	Labour costs are affected by regional minimum wage policies and labour market supply and demand, with the fluctuation range typically being ±10–15%.

Supplementary References

1 Sands, J. M., Blount, M. A. & Klein, J. D. Regulation of renal urea transport by vasopressin. *Trans. Am. Clin. Climatol. Assoc.* **122**, 82 (2011).

2 Stumpe, M. C. & Grubmüller, H. Aqueous urea solutions: structure, energetics, and urea aggregation. *Phys. Chem. B* **111**, 6220-6228 (2007).

3 Li, P. *et al.* Hydrogen bond network connectivity in the electric double layer dominates the kinetic pH effect in hydrogen electrocatalysis on Pt. *Nat. Catal.* **5**, 900-911 (2022).

4 Kim, Y., Han, H. & Shin, H.-J. Controlled Cooperativity of Proton Tunneling in a Water Trimer. *Nano Letters* (2025).

5 Chen, T. *et al.* Saline water treatment coupled with carbon dioxide capture by bipolar membrane electrodialysis in a continuous feed-bleed mode: The effect of proton leakage. *Chem. Eng. J.* **498**, 155092 (2024).

6 Markvoort, A. J., Hilbers, P. & Nedea, S. Molecular dynamics study of the influence of wall-gas interactions on heat flow in nanochannels. *Phys. Rev. E Stat. Nonlin. Soft. Matter. Phys.* **71**, 066702 (2005).

7 Zhou, L., Zhu, J., Zhao, Y. & Ma, H. A molecular dynamics study on thermal conductivity enhancement mechanism of nanofluids–Effect of nanoparticle aggregation. *Int. J. Heat Mass Tran.* **183**, 122124 (2022).

8 Naughton, J. W. & Sheplak, M. Modern developments in shear-stress measurement. *Prog. Aerosp. Sci.* **38**, 515-570 (2002).

9 Allen, J. P. *Biophysical Chemistry*. (John Wiley & Sons, 2009).

10 Sohrab, S. H. The invariant planck energy distribution law and its connection to the Maxwell-Boltzmann distribution function. *WSEAS Transactions on Mathematics* **6**, 254 (2007).

11 Valdez, S. I., Hernández, A. & Botello, S. A Boltzmann based estimation of distribution algorithm. *Inform. Sci.* **236**, 126-137 (2013).

12 Huber, D. & Van Vleck, J. The role of Boltzmann factors in line shape. *Rev. Mod.*

Phys. **38**, 187 (1966).

13 Matta, C. F., Hernández-Trujillo, J., Tang, T. H. & Bader, R. F. Hydrogen–hydrogen bonding: a stabilizing interaction in molecules and crystals. *Chem. Eur. J.* **9**, 1940-1951 (2003).

14 Shi, B. *et al.* Short hydrogen-bond network confined on COF surfaces enables ultrahigh proton conductivity. *Nat. Commun.* **13**, 6666 (2022).

15 Wood, P. A., McKinnon, J. J., Parsons, S., Pidcock, E. & Spackman, M. A. Analysis of the compression of molecular crystal structures using Hirshfeld surfaces. *CrystEngComm* **10**, 368-376 (2008).

16 Israelachvili, J. N. *Intermolecular and surface forces*. (Academic Press, 2011).

17 Goto, Y., Takahashi, N. & Fink, A. L. Mechanism of acid-induced folding of proteins. *Biochemistry* **29**, 3480-3488 (1990).

18 Vu, T., Weaver, M. R., Kasting, G. B. & Koenig, P. Effect of pH on the structure and dynamics of wormlike micelles in an amino acid-derived surfactant composition. *Langmuir* **37**, 4112-4120 (2021).

19 Aveyard, R. & Haydon, D. A. *An introduction to the principles of surface chemistry*. (Cambridge University Press, 1973).

20 Parsegian, V. A. *Van der Waals forces: a handbook for biologists, chemists, engineers, and physicists*. (Cambridge University Press, 2005).

21 Yang, H.-R. *et al.* Advanced electrochemical membrane technologies for near-complete resource recovery and zero-discharge of urine: Performance optimization and evaluation. *Water Res.* **263**, 122175 (2024).

22 Raghuvanshi, S. *et al.* Dual control on structure and magnetic properties of Mg ferrite: role of swift heavy ion irradiation. *J. Magn. Magn. Mater.* **471**, 521-528 (2019).

23 Abramova, E., Lapides, I. & Yariv, S. Thermo-XRD investigation of monoionic montmorillonites mechanochemically treated with urea. *J. Therm. Anal. Calorim.* **90**, 99-106 (2007).

24 Atahar, A., Mafy, N. N., Rahman, M. M., Mollah, M. Y. A. & Susan, M. A. B. H.

Aggregation of urea in water: Dynamic light scattering analyses. *J. Mol. Liq.* **294**, 111612 (2019).

25 Jayaprakash, R. & Kumaradass, P. Growth and Characterization of Urea L-valine a Organic Non Linear Optical Crystal. *Orient. J. Chem.* **29**, 1409 (2013).

26 Sarigul, N., Korkmaz, F. & Kurultak, İ. A new artificial urine protocol to better imitate human urine. *Sci. Rep.* **9**, 20159 (2019).

27 Simha, P., Courtney, C. & Randall, D. G. An urgent call for using real human urine in decentralized sanitation research and advancing protocols for preparing synthetic urine. *Front. Environ. Sci.* **12**, 1367982 (2024).
