# Supplementary material for: Synthetic urine oversimplification results in misleading membrane fouling mechanisms in bipolar membrane electrodialysis
Source: Nat Commun. 2026 Apr 10;17:3395. doi: 10.1038/s41467-026-70034-w (PMC13069111; doi:10.1038/s41467-026-70034-w)
Supplement: Supplementary file 2 — Reporting summary [file 41467_2026_70034_MOESM2_ESM.pdf]

nature portfolio

Corresponding author(s): DAPR NCOMMS-25-42964

Last updated by author(s): Jan 17, 2026

Reporting Summary

Nature Portfolio wishes to improve the reproducibility of the work that we publish. This form provides structure for consistency and transparency in reporting. For further information on Nature Portfolio policies, see our Editorial Policies and the Editorial Policy Checklist.

Statistics

For all statistical analyses, confirm that the following items are present in the figure legend, table legend, main text, or Methods section.

n/a	Confirmed	
☐	☒	The exact sample size (*n*) for each experimental group/condition, given as a discrete number and unit of measurement
☐	☒	A statement on whether measurements were taken from distinct samples or whether the same sample was measured repeatedly
☒	☐	The statistical test(s) used AND whether they are one- or two-sided<br>*Only common tests should be described solely by name; describe more complex techniques in the Methods section.*
☐	☒	A description of all covariates tested
☒	☐	A description of any assumptions or corrections, such as tests of normality and adjustment for multiple comparisons
☐	☒	A full description of the statistical parameters including central tendency (e.g. means) or other basic estimates (e.g. regression coefficient) AND variation (e.g. standard deviation) or associated estimates of uncertainty (e.g. confidence intervals)
☒	☐	For null hypothesis testing, the test statistic (e.g. *F*, *t*, *r*) with confidence intervals, effect sizes, degrees of freedom and *P* value noted<br>*Give P values as exact values whenever suitable.*
☒	☐	For Bayesian analysis, information on the choice of priors and Markov chain Monte Carlo settings
☒	☐	For hierarchical and complex designs, identification of the appropriate level for tests and full reporting of outcomes
☐	☒	Estimates of effect sizes (e.g. Cohen's *d*, Pearson's *r*), indicating how they were calculated

Our web collection on statistics for biologists contains articles on many of the points above.

Software and code

Policy information about availability of computer code

Data collection	Material Studio 2019
Data analysis	Origin 2024, Avantage6.9, Microsoft PowerPoint 2021 and Material Studio 2019

For manuscripts utilizing custom algorithms or software that are central to the research but not yet described in published literature, software must be made available to editors and reviewers. We strongly encourage code deposition in a community repository (e.g. GitHub). See the Nature Portfolio guidelines for submitting code & software for further information.

Data

Policy information about availability of data

All manuscripts must include a data availability statement. This statement should provide the following information, where applicable:

- Accession codes, unique identifiers, or web links for publicly available datasets
- A description of any restrictions on data availability
- For clinical datasets or third party data, please ensure that the statement adheres to our policy

The data supporting the findings of this study are available within the paper and its Supplementary Information. Additional data are available from the corresponding authors upon reasonable request. Source data are provided with this paper.

Research involving human participants, their data, or biological material

Policy information about studies with human participants or human data. See also policy information about sex, gender (identity/presentation), and sexual orientation and race, ethnicity and racism.

Reporting on sex and gender	n/a
Reporting on race, ethnicity, or other socially relevant groupings	n/a
Population characteristics	n/a
Recruitment	n/a
Ethics oversight	n/a

Note that full information on the approval of the study protocol must also be provided in the manuscript.

Field-specific reporting

Please select the one below that is the best fit for your research. If you are not sure, read the appropriate sections before making your selection.

☐ Life sciences ☐ Behavioural & social sciences ☒ Ecological, evolutionary & environmental sciences

For a reference copy of the document with all sections, see nature.com/documents/nr-reporting-summary-flat.pdf

Ecological, evolutionary & environmental sciences study design

All studies must disclose on these points even when the disclosure is negative.

Study description	This study employed a sequential batch experiment to evaluate a single factor: synthetic urine composition. The factor had four levels (Groups A, B, C, D), each representing a distinct formulation with graded complexity. Each formulation group was processed serially in a single, custom-built BMED membrane stack over seven consecutive operational batches. Therefore, the entire sequence of seven batches for a given formulation constitutes a single, longitudinal experimental unit. The design is best described as a single-factor, repeated-measures (over time/batches) study.
Research sample	The research samples were synthetic urine formulations meticulously prepared in the laboratory. Their compositions were based on a comprehensive analysis of urban urine composition (Supplementary Table 1). The samples were defined as:<br>Group A: A conventional simplified formulation containing only dominant inorganic salts and urea.<br>Group B: An expanded formulation incorporating Group A components plus core small organic metabolites (creatinine and uric acid).<br>Group C: A complex formulation further introducing the macromolecular organic model protein Bovine Serum Albumin (BSA).<br>Group D: An inorganic-only formulation, excluding all organic components.<br>These samples were chosen to deconstruct and represent the key constituent categories (ions, small organics, macromolecules) of real human urine, allowing for a systematic investigation of their individual and synergistic effects on the BMED process.
Sampling strategy	No statistical method was used to predetermine sample size, and there was no parallel replication of the entire BMED system. The sample size was defined as a sequence of seven consecutive operational batches for each of the four formulation groups (A-D). This sample size (number of batches) was chosen to be consistent with established protocols in membrane fouling studies and was sufficient to capture the longitudinal trends of performance decay and fouling evolution. The experimental design thus constitutes a repeated-measures study over time (batches) within each system, rather than a parallel-group comparison.
Data collection	Data collection was conducted during and after BMED operation. System performance data (solution pH, conductivity, ion concentrations via ion chromatography, urea concentration via a colorimetric method) were collected in real-time or from sampled solutions from corresponding chamber. Membrane characterization data were collected post-operation from samples taken from the used membrane stack using the following instruments: surface morphology and elemental composition by SEM-EDS (MIRA LMS, Tescan), surface chemical composition by ATR-FTIR (Nicolet iS20, Thermo) and XPS (K-Alpha, Thermo), and surface wettability by Contact Angle Goniometer (SDC 200S, SINDIN). Theoretical data were obtained from Molecular Dynamics simulations performed in the FORCITE module of Materials Studio. All data were collected by the authors.
Timing and spatial scale	Data collection was conducted in a controlled laboratory environment. For each formulation group, a sequence of seven batch experiments was run consecutively, with each batch lasting 2 hours. The spatial scale was confined to a single, lab-scale BMED membrane stack, with each chamber having a volume of 0.15 L and membranes with an effective area of 55 cm2.
Data exclusions	No data were excluded from the analyses. All collected data from the seven batches for all groups are reported.
Reproducibility	The reproducibility of the observed trends is demonstrated by the consistent and quantifiable performance decay and fouling mechanisms observed over the seven sequential batches for each formulation. Furthermore, the experimental findings were robustly corroborated by multiple, complementary characterization techniques (SEM-EDS, FTIR, XPS, MD simulations), which all converged on

the same mechanistic conclusions. All experimental procedures were carried out successfully without failure.

Randomization	Randomization was not used in this study. The investigation required a systematic, sequential comparison of specific urine compositions to understand their time-dependent (batch-to-batch) effects on fouling. The order of testing the formulation groups was predetermined. All operational parameters (applied voltage, flow rate, initial volumes) were rigorously controlled and kept identical across all experimental sequences to isolate and assess the effect of the urine composition.
Blinding	Blinding was not used during data collection and analysis. This was not feasible due to the nature of the sequential experimental protocol, where the specific formulation being tested was known to the researchers during system operation and subsequent membrane analysis. However, the primary datasets consist of quantitative instrument readings and computational outputs, which are objective and minimize the potential for observer bias.

Did the study involve field work? ☐ Yes ☒ No

Reporting for specific materials, systems and methods

We require information from authors about some types of materials, experimental systems and methods used in many studies. Here, indicate whether each material, system or method listed is relevant to your study. If you are not sure if a list item applies to your research, read the appropriate section before selecting a response.

Materials & experimental systems

n/a	Involved in the study
☒	☐ Antibodies
☒	☐ Eukaryotic cell lines
☒	☐ Palaeontology and archaeology
☒	☐ Animals and other organisms
☒	☐ Clinical data
☒	☐ Dual use research of concern
☒	☐ Plants

Methods

n/a	Involved in the study
☒	☐ ChIP-seq
☒	☐ Flow cytometry
☒	☐ MRI-based neuroimaging

Plants

Seed stocks	n/a
Novel plant genotypes	n/a
Authentication	n/a
